# Synthesis and Conformational Analysis of Fluorinated Uridine Analogues Provide Insight into a Neighbouring-Group Participation Mechanism

**DOI:** 10.3390/molecules25235513

**Published:** 2020-11-25

**Authors:** Freideriki Michailidou, Tomas Lebl, Alexandra M. Z. Slawin, Sunil Vishnuprasadji Sharma, Murray J. B. Brown, Rebecca Jane Miriam Goss

**Affiliations:** 1 EaStCHEM School of Chemistry, University of St Andrews, North Haugh, St Andrews, Fife KY16 9ST, UK; fm59@st-andrews.ac.uk (F.M.); tl12@st-andrews.ac.uk (T.L.); amzs@st-andrews.ac.uk (A.M.Z.S.); svs4@st-andrews.ac.uk (S.V.S.); 2 GlaxoSmithKline, Stevenage SG1 2NY, UK; murray.j.brown@gsk.com

**Keywords:** nucleoside, fluorine, fluorination, neighbouring-group participation, mechanism

## Abstract

Fluorinated nucleoside analogues have attracted much attention as anticancer and antiviral agents and as probes for enzymatic function. However, the lack of direct synthetic methods, especially for 2′,3′-dideoxy-2′,3′-difluoro nucleosides, hamper their practical utility. In order to design more efficient synthetic methods, a better understanding of the conformation and mechanism of formation of these molecules is important. Herein, we report the synthesis and conformational analysis of a 2′,3′-dideoxy-2′,3′-difluoro and a 2′-deoxy-2′-fluoro uridine derivative and provide an insight into the reaction mechanism. We suggest that the transformation most likely diverges from the S_N_1 or S_N_2 pathway, but instead operates via a neighbouring-group participation mechanism.

## 1. Introduction

**Scheme 1:**
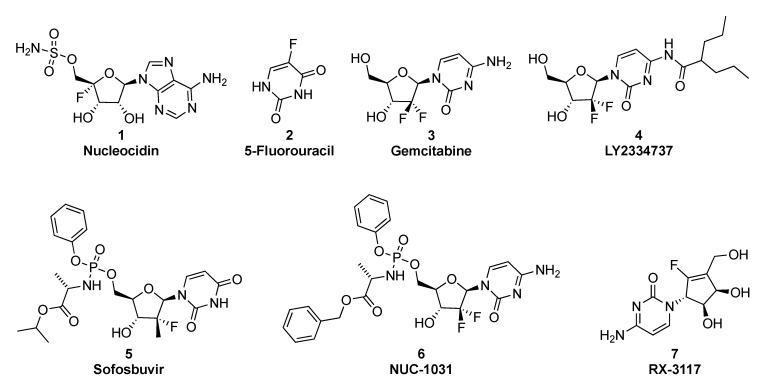
Fluorine moieties present in nucleocidin, 5-fluorouracil, gemcitabine, LY2334737, sofosbuvir, NUC 1031 and RX-3117.

Fluorinated nucleosides and nucleotide analogues have attracted much attention as antiviral and anticancer drugs or mechanistic probes [1,2,3,4]. Since the development of the first fluorine-containing drug in 1957, the research in the field of fluorine chemistry has flourished, leading to noteworthy industrial applications [5]. To date, the portfolio of fluorinated drugs includes over 150 compounds, constituting ~20% of all pharmaceuticals (increasing to well above 30% for recent FDA-approved drugs) [5]. Furthermore, the widely used antidepressant fluoxetine (Prozac), the antibacterial ciprofloxacin (Ciprobay) and the anti-inflammatory fluticasone are fluorine-containing drugs. A selection of the most successful organofluorine-containing blockbuster drugs, including sitagliptin (for the treatment of diabetes); sofosbuvir (hepatitis C); dolutegravir, bictegravir and efavirenz (HIV); and enzalutamide (prostate cancer), highlight the significance of fluorinated bioactive compounds [6,7]. Fluorinated nucleoside analogues nucleocidin **1**, 5-fluorouracil **2**, gemcitabine **3**, LY2334737 **4**, sofosbuvir **5**, NUC-1031 **6** and RX3117 **7** are shown in Scheme 1 [8,9]. Nucleocidin, a natural product and trypanosome-active antibiotic, was initially isolated from *Streptomyces calvus* and bears a fluorine atom at the 4′ position [9]. 5-Fluorouracil is an anticancer agent manufactured on an industrial scale, while gemcitabine, which contains two fluorine atoms at the 2′ position, is a chemotherapy medication used to treat a number of types of cancer [8,10]. LY2334737 was designed as a prodrug of gemcitabine, with anticipated increased bioavailability; however, it was discontinued due to its hepatic toxicity in patients [11,12]. Sofosbuvir is a medication for hepatitis C, while NUC-1031 is a 5′-phosphoramidate prodrug of gemcitabine [13,14]. RX-3117 has shown efficacy in clinical trials against drug-resistant tumours and has received “orphan drug designation” for pancreatic cancer from the U.S. Food and Drug Administration (FDA) and from the European Commission (EC) [15,16].

The unique steric and electronic properties of fluorine have led to its use in probing enzymatic mechanisms and to the C-F bond playing a very dominant role in medicinal chemistry [5,17,18,19,20]. As a close steric replacement for hydrogen, fluorine can introduce important electronic changes in a molecule with minimal steric perturbation. Fluorine is also used as a hydroxyl mimic, given its hydrogen bonding ability and the proximity of the C-F bond length (1.35 Å) to the C-O bond length (1.43 Å) [17]. Due to the stability of the C-F bond (105.4 kcal mol^−1^), replacing a hydrogen or a hydroxyl group with fluorine significantly improves the metabolic stability of the compound. Oxidative degradation of biomolecules in vivo, for example oxidation by P450 enzymes, can be prevented via the utilisation of fluorinated analogues [5,21,22]. The basicity of a molecule can also be reduced by introducing a fluorine atom in proximity to the basic group [21,22]. Notably, incorporating one or more C-F bonds within a molecule provides a means of increasing lipophilicity and improving bioavailability as well as promoting hydrophobic interactions between the drug and binding sites on receptors or enzymes [18].

The design of new fluorinated nucleoside analogues and the need for their syntheses have driven the research in the field of fluorine nucleoside chemistry [23]. Owing to the aforementioned unique properties of fluorine, fluorinated nucleoside analogues have found applications as chemotherapeutics in the clinic, and in general as drugs and mechanistic probes. Replacement of one or more hydroxyls of the nucleoside ring with an almost isosteric C-F bond impacts the electronics and the hydrophobicity of the compounds as well as reducing their ability to hydrogen bond. Furthermore 2′,3′-dideoxy nucleosides cannot be extended within a natural nucleic acid polymer, and as well as inhibiting enzymes such as reverse transcriptase, they can be used to terminate replication or transcription/reverse transcription of such a polymer [1]. Fluorination at C2′ has been long been known to both promote furanose conformation change and improve metabolic stability [24], whilst replacement of the 3′ and 5′ alcohols prevents chain extension [25]. Such modified nucleic acids can also inhibit enzymes such as DNA or RNA polymerases, reverse transcriptases and nucleoside phosphorylases that are essential for cancer cell growth and viral replication [26,27].

However, introducing a fluorine atom into a sugar moiety in nucleosides can be challenging. For the synthesis of 2′,3′-dideoxy-2′,3′-difluoro nucleosides, the potential for hydroxyl elimination rather than substitution, due to the poor nucleophilicity yet considerable basicity of fluoride, adds to the synthetic challenge. A recent four-step sequence [28] giving access to series of nucleoside analogues, though notably no ribose-fluorinated analogues, was reported using a “ribose-last” approach whilst we were preparing this manuscript for publication; in a complementary study, we set out to explore a direct approach to ribose-fluorinated 5′-*O*-protected uridine analogues to gain greater insight into their conformations and thereby the mechanisms by which they might be formed.

It has been reported that electronegative substituents at the C2′ of adenosines favour the C3′ endo-conformation, placing the hydroxyl diaxial to an acidic proton and promoting E2 elimination. However, bulky substituents at C2′ and C5′ mostly favour a C2′ endo-conformation, preventing elimination [29,30]. Rationalising in this paper that a large protecting group could be used to promote substitution rather than elimination, we explored the potential for the direct nucleophilic fluorination of the 2′ and 3′ positions in 5′-*O*-protected uridine analogues. We further performed structural and conformational analysis of the derived uridine analogues and provided insight into the reaction mechanism.

## 2. Results

### 2.1. Synthesis of Fluorinated Nucleoside Analogues

**Scheme 2:**
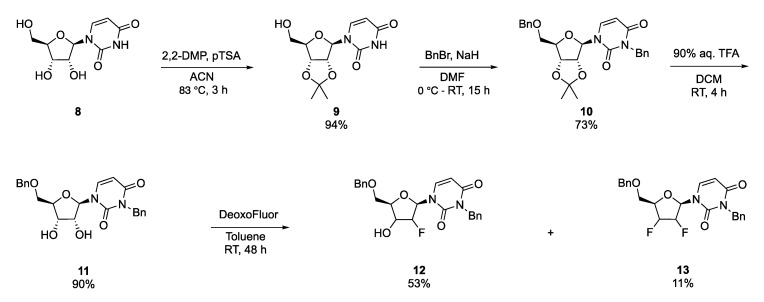
Benzylation and fluorination reactions leading to compounds **12** and **13**. Abbreviations are as following: 2,2-DMP: 2,2-dimethoxypropane; ACN: acetonitrile; BnBr: benzyl bromide; NaH: sodium hydride; TFA: trifluoroacetic acid, DCM: dichloromethane; DeoxoFluor: bis(2-methoxyethyl)aminosulfur trifluoride

Unlike previously reported strategies that involve a two-step insertion of fluorine in the 2′ and 3′ positions of the nucleoside for the synthesis of the 2,3-dideoxy-2,3-difluoro sugars followed by base condensation [31,32], we aimed at the direct functionalisation of the corresponding positions in 5′-*O*-protected uridine analogues. Whilst our initial attempts to functionalise the 2′ and 3′ hydroxyl groups of 5′-*O*-carboxybenzyl (Cbz) and of 5′-*O*-benzoyl (Bz) uridine derivatives using fluorinating reagents diethylaminosulfur trifluoride (DAST) and Deoxo-Fluor proved unsuccessful, we attempted the utilisation of analogues protected by benzyl (Bn) groups. Bn groups are often used in nucleoside and carbohydrate chemistry as being compatible with a wide range of reaction conditions, as they are relatively stable to both acid and base [33], and would potentially be stable to the demanding reaction conditions and long reaction times required for fluorination. Acetonide **9**, which was synthesised using reported protocols [34], was further subjected to treatment with benzyl bromide and sodium hydride to yield compound **10** in a 73% yield. Standard deprotection using aqueous trifluoroacetic acid (TFA) removed the acetonide function and yielded **11**, the starting material for the fluorination reaction. Treatment of **11** with Deoxo-Fluor (10 equiv.) in toluene at RT for 48 h led to compounds **12** and **13** (Scheme 2). The desired monofluorinated product **12** was obtained in a 53% yield after flash column chromatography. The ^19^F NMR revealed a singlet at −185 ppm, while the proton coupled spectrum revealed a ddd with the expected coupling constants: ^2^*J*_F-H2′_ 48.8 Hz, ^3^*J*_F-H1′_ 20.9 Hz, ^3^*J*_F-H3′_ 9.9 Hz. The position of the fluorine at the C2′ was verified by ^1^H NMR, ^13^C NMR and ^1^H{F}NMR (SI, Figures S1–S4). The apparent singlet at 5.92 ppm in the fluorine-decoupled ^1^H NMR corresponds to the H1′, and splits into a doublet with a coupling constant of 20.9 Hz in the fluorine-coupled spectrum. This coupling constant is representative of ^3^*J* proton–fluorine interaction. The ^19^F{H}NMR of the compound assigned as the difluorinated molecule **13**, revealed the two doublets at −192 and −208 ppm (^3^*J* = 14.3 Hz). Further details of NMR analysis are provided in the SI (Figures S1–S4).

### 2.2. Conformational Analysis of the Synthesized Analogues

The sugar moiety of the nucleosides is found with four different stereochemistries: ‘*ribo*’, where the substituents on both C2′ and C3′ are in a ‘down’ configuration; ‘*ara*’, where the C2′ has the substituent ‘up’; ‘*xylo*’, where the C2′ substituent is ‘down’ and the 3′ is ‘up’; and ‘*lyxo*’, where both substituents are ‘up’ (Scheme S1A). Electronegative atoms at C2′ are known to reduce the magnitude of *J*_H1′-H2′_ and *J*_H2′-H3′_ [35]. However, the 2′-fluoro-β-d-dideoxyuridine molecules are reported with a coupling constant of 3.3 Hz for H1′-H2′ [36] and in general, J_H1′-H2′_ in β-nucleosides with a 2′-ara substituent is in the range of 3–4 Hz [37]. Barchi et al. [38] reported the synthesis and conformational analysis of the 2′,3′-dideoxy-2′,3′-difluoro uridine analogues with the *ara*- and *xylo*-stereochemistry (Table S1), and the general trend of the values of the coupling constants mentioned above are in agreement with McAtee et al. [37].

The absence of a *J*_H1′-H2′_ coupling in the ^1^H{F}NMR of **12** suggested that F2′ was more likely to be pointing ‘down’ (*xylo*- or *ribo*-stereochemistry). However, not all the data in the literature seem to follow this general trend; the reason for this might be the tendency of nucleosides to exist in two distinct conformations: the so-called *S*-type (south, C2′-endo) and *N*-type (north, C3’-endo) conformation (Scheme S1B). It is reported that for each diastereomer, two potential conformers can be observed and experimental values of the H-H and F-H coupling constants would be influenced [35]. In order to verify the stereochemistry of the derived structures, we performed a further investigation and utilisation of the heteronuclear NOE experiments. This technique has made a significant contribution to the conformational analysis of nucleosides and it is very often utilised for fluorinated nucleosides [39,40,41]. Heteronuclear NOE experiments and Density-functional theory (DFT) calculations were then performed (B3LYP/6-31G* level of theory) to determine the relative stereochemistry at the 2′- and 3′-positions of compound **12**.

**Figure 1:**
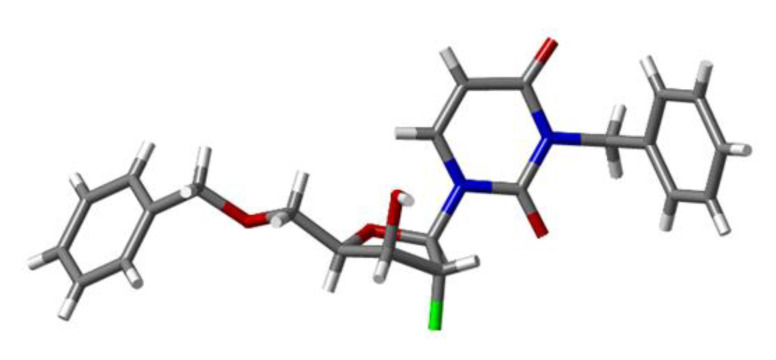
X-ray crystal structure of molecule **12**.

The eight conformers (the north and south conformer for each of the diastereomers *SR*, *RR*, *RS* and *SS*) are shown in the SI (Figure S6). In Table 1 and Table 2, the theoretically calculated values of the coupling constants [42,43] and the distances of each proton of the sugar moiety from the fluorine are shown for each conformer. Comparison of those estimated values with the experimental data led to the conclusion that the C1 conformer (*RS*-north) was the closest model to our observations, suggesting a retention of stereochemistry at C2′ (C1, Table 1 and Table 2). Furthermore, ring conformation 1 (north) is predicted to be more stable for all diastereomers apart from D (*SS*); in the case of C (*RS*), the energy difference is quite substantial. The ring is quite rigid and C1 will be the predominant conformer, so any conformational equilibrium can be neglected. Later, X-ray crystallography allowed for a complete stereochemical assignment (Figure 1) and confirmed the results of NMR analysis.

**Figure 2:**
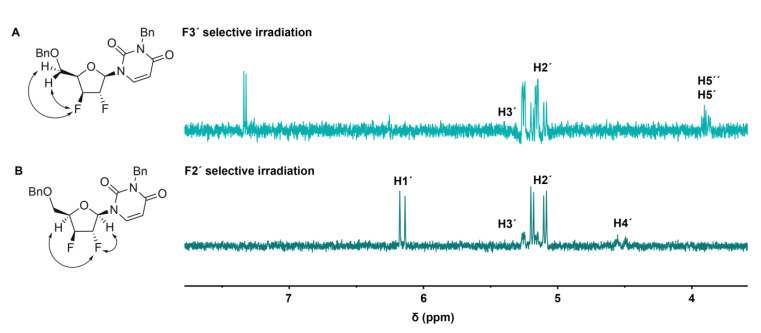
Heteronuclear NOE experiments (HOESY) for compound **13** (282 MHz, CDCl_3_). The mixing time of the experiment was 600 ms. (**A**) Selective irradiation of the F3′. (**B**) Selective irradiation of the F2′.

Investigation of the stereochemistry of molecule **13** was then performed by a series of selective heteronuclear NOE experiments (HOESY). ^1^H, ^19^F HMBC confirmed that the doublet at −192 ppm corresponds to F2′ and the doublet at −208 ppm to F3′ (SI, Figure S5). The selective HOESY allowed the determination of the stereochemistry. Selective irradiation of the F3′ (Figure 2A) revealed a strong NOE with the H5′ and H5″, which indicates that the F3′ is in the β-face of the sugar. Selective irradiation of the F2′ (Figure 2B) led to a strong NOE on the H1′ and a weak NOE on the H4′, which, combined with the previous experiments, suggests that the F2′ is pointing in the opposite direction.

### 2.3. Mechanistic Proposal: Neighbouring-Group Participation

**Scheme 3:**
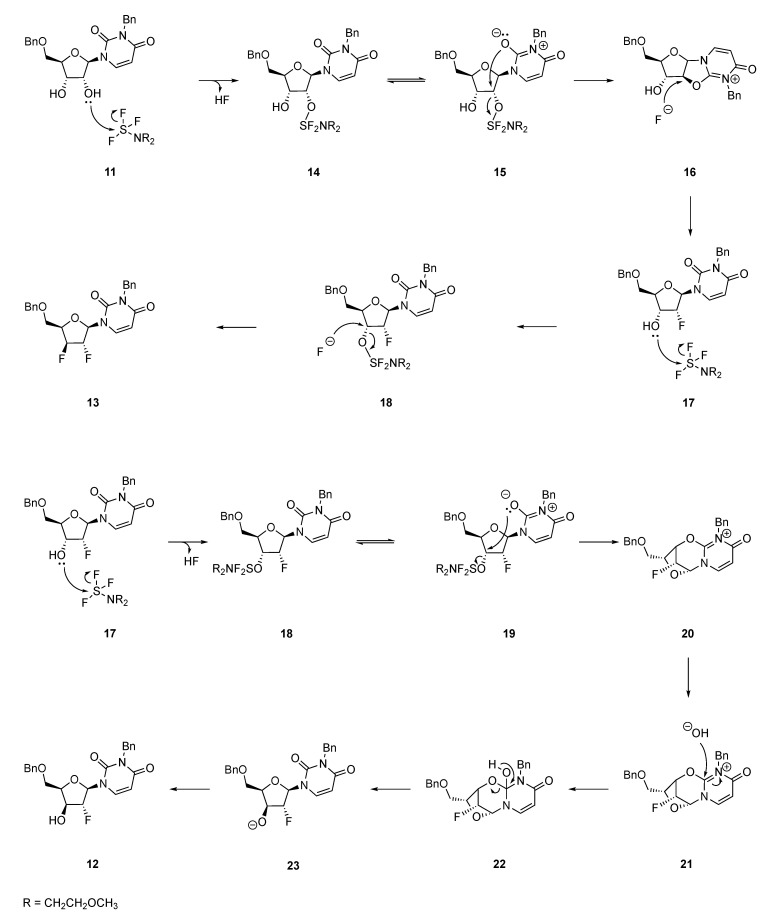
Proposed mechanism leading to compounds **12** and **13**.

As suggested by the NOE and crystallographic data, the stereochemistry for compounds **12** and **13** is suggested to be *xylo*. For a simpler system, such as an alcohol or diol, an inversion of the stereochemistry at C2′ due to an S_N_2 mechanism would be expected. On the contrary, the retention of stereochemistry observed in this case could be contributed to the ‘neighbouring-group participation’, an established phenomenon with sugars and nucleosides. Indeed, the formation of anhydro nucleosides [44] such as **16** and **20** during DAST-type reactions has been previously reported [41,45,46], and an adapted mechanistic proposal is shown in Scheme 3. We propose that the benzyl- as an electron-donating group is postulated to have a significant impact on the mechanistic outcome of the chemical transformation.

We suggest that the reaction initially occurs with a nucleophilic attack of the ribose O2′ to the electrophilic sulphur of the Deoxo-Fluor reagent. Then, the lone pair of the oxygen of the uracil moiety attacks the C2′ of the ribose, leading to the first inversion of stereochemistry; the excellent leaving group is removed and the anhydro nucleoside **16** is formed. The fluoride anion attacks the C2′, and this second inversion leads to the fluorinated nucleoside with the retained stereochemistry.

## 3. Discussion

A method for the fluorination of 2′ and 3′ position of uridine derivatives has been developed, allowing for the structural and conformational analysis of these compounds. Nuclear Overhauser NMR techniques and X-ray crystallography have allowed the full structural and stereochemical assignment of the synthetic nucleoside analogues. The deoxy-fluorination reaction that allowed us to obtain our desired targets led to a retention of stereochemistry on the C2′ and an inversion at C3′, an interesting twist that yielded the *xylo*-derivatives. This twist can be attributed to neighbouring-group participation effects, common for uridine derivatives. This effect has been previously reported to contribute to the formation of anhydro nucleosides [41,44,45,46]. The relative stereochemistry of the F2′ on the monofluorinated molecules was assigned by NOE spectroscopy as well as comparison of the theoretical and experimental values for *J*_F-H1′_ and *J*_H1′-H2′_. The final confirmation of the absolute stereochemistry was accomplished by X-ray crystallography. For the difluorinated analogues, HOESY spectroscopy gave us insight into examining the stereochemistry. Selective irradiation of the F2′ revealed a weak NOE with H4′ and a strong NOE with H1′, while irradiation of F3′ revealed an NOE with H5′ and H5′’, which suggests that the compound has the *xylo*- stereochemistry. We proposed that the mechanistic and conformational insight provided by this study can guide the further development of novel synthetic methods for the synthesis of the highly valuable, yet synthetically challenging fluorinated nucleosides.

## 4. Materials and Methods

### 4.1. General Considerations

All starting materials, reagents and solvents were commercially available and were used without further purification, unless otherwise stated. Chemical reagents and solvents were bought from Sigma Aldrich (Dorset, UK), Alfa Aesar (Cheshire, UK), Fisher (Loughborough, UK) or Fluorochem (Glossop, UK). Reactions were carried out under nitrogen or argon atmosphere in oven-dried glassware. When not specified, reactions were performed in clean, air-dried glassware. Fluorination reactions were carried out in oven-dried Teflon flasks. Thin layer chromatography (TLC) was performed using Machery Nagel (Dylan, Germany) polyester-backed sheets coated with silica (and fluorescent indicator UV254) to a thickness of 0.20 mm. The reactions were monitored by TLC, and the plates were visualised under a UV lamp (254/365 nm, Mineralight model UVGL-58 lamp) and stained with potassium permanganate. Melting point was recorded using a Stuart SMP10 melting point apparatus and is uncorrected. NMR spectra of synthesised molecules were recorded at room temperature (RT) using Bruker Avance 500 (^1^H at 500 MHz, ^13^C at 126 MHz, ^19^F at 470 MHz) and Bruker Avance 400 (^1^H at 400 MHz, ^13^C at 101 MHz) instruments, using deuterated solvents (Billerica, MA, USA). Chemical shifts, *δ*, are reported in parts per million (ppm) and are quoted relative to the centre of the reference nondeuterated solvent peak for *δ*_H_ (CDCl_3_: 7.26 ppm; CD_3_OD: 4.87, 3.31 ppm) and *δ*_C_ (CDCl_3_: 77.16 ppm; CD_3_OD: 49.00 ppm). ^13^C NMR spectra were recorded with ^1^H decoupling, while all spectra were processed and analysed using MestReNova 8.0.1 (Mestrelab Research, St. Louis, MO, USA). Coupling constants *J* are given in hertz (Hz). Multiplicity patterns are described as follows: bs—broad singlet, s—singlet, t—triplet, tt—triplet of triplets, ddd—doublet of doublets of doublets, ddt—doublet of doublets of triplets, m—multiplet. One-dimensional ^1^H, ^13^C DEPTQ (distorsionless enhancement by polarization transfer including the detection of quaternary nuclei), ^19^F experiments as well as two-dimensional [^1^H, ^1^H] DQFCOSY (double quantum filtered correlation spectroscopy), [^1^H, ^13^C] HSQC (heteronuclear single quantum correlation spectroscopy), and [^1^H, ^13^C] HMBC (heteronuclear multiple bond correlation spectroscopy) experiments were utilised for structure determination.

### 4.2. Preparation of 2′,3′-O-Isopropylidene Uridine (**9**)

Uridine **8** (500 mg, 2 mmol) was dissolved in acetonitrile (30 mL). 2,2-dimethoxypropane (2 mL, 16 mmol) and *p*-toluenesulfonic acid (15 mg) were added and the mixture was refluxed for 3 h. The reaction mixture was concentrated under vacuum to give a brown residue. The residue was purified by flash chromatography (100% DCM to 100% acetone in a stepwise gradient) to give the desired compound **9**. [47] (534 mg, 94% yield). ^1^H-NMR (CD_3_OD, 500 MHz) *δ* (ppm) 7.86 (d, *J =* 8.1 Hz, 1H), 5.89 (d, *J =* 3.0 Hz, 1H), 5.71 (d, *J =* 8.1 Hz, 1H), 4.93 (dd, *J =* 6.6, 3.2 Hz, 1H), 4.84 (dd, *J =* 6.5, 3.4 Hz, 1H), 4.23 (m, 1H), 3.79 (dd, *J =* 12.0, 3.6 Hz, 1H), 3.73 (dd, *J =* 12.2, 3.8 Hz, 1H), 1.57 (s, 3H), 1.38 (s, 3H). ^13^C-NMR-DEPTQ (CD_3_OD, 126 MHz) *δ* (ppm) 164.8, 150.6, 142.4, 113.7, 101.2, 92.7, 86.9, 84.4, 80.8, 61.6, 26.1, 24.1. MS (ES^+^) *m*/*z* 285.1 ([M + H]^+^, 100%), 569.2 ([2M + H]^+^, 67%), HRMS (ES^+^) *m*/*z* calc. for C_12_H_17_N_2_O_6_ [M + H]^+^ 285.1081, found 285.1075.

### 4.3. Preparation of 3,5′-dibenzyl-2′,3′-O–Isopropylidene Uridine (**10**)

NaH (60% dispersion in oil, 260 mg, 14.1 mmol) was added into a stirred solution of 2′,3′-*O*-isopropylidene uridine **9** (1 g, 3.52 mmol) in dry DMF (25 mL) under argon atmosphere, and the mixture was cooled at 0 °C using an ice bath. BnBr (24.65 mmol, 2.93 mL) was added dropwise under argon atmosphere, with cooling and stirring. The reaction was monitored by TLC (hexane/acetone: 2/1) and judged complete after 4 h. The reaction mixture was quenched with MeOH (25 mL) and concentrated and coevaporated with toluene (50 mL). The crude material was purified by flash column chromatography (hexane/acetone: stepwise gradient) to give the desired product **10** (1.2 g, 73% yield) as a white solid. ^1^H NMR (CDCl_3_, 500 MHz) *δ* (ppm) 7.52 (d, *J* = 8.1 Hz, 1H), 7.46 (apd, *J* = 7.3 Hz, 2H), 7.36–7.23 (m, 8H), 5.93 (d, *J* = 2.6 Hz, 1H), 5.55 (d, *J* = 8.1 Hz, 1H), 5.11 (d, *J* = 13.7 Hz, 1H), 5.02 (d, *J* = 13.8 Hz, 1H), 4.81 (dd, *J* = 6.2, 2.7 Hz, 1H), 4.73 (dd, *J* = 6.2, 2.6 Hz, 1H), 4.54–4.48 (m, 2H), 4.46–4.30 (m, 1H), 3.76 (dd, *J* = 10.6, 2.5 Hz, 1H), 3.65 (dd, *J* = 10.6, 3.5 Hz, 1H), 1.58 (s, 3H), 1.35 (s, 3H). ^13^C NMR (CDCl_3_, 126 MHz) *δ* (ppm) 162.8, 151.0, 138.7, 137.2, 136.8, 129.3, 128.7, 128.5, 128.3, 128.0, 127.7, 114.1, 101.7, 93.6, 85.9, 85.6, 81.1, 73.9, 70.3, 44.2, 27.4, 25.5. MS (ES^+^) *m/z* 465.2 ([M + H]^+^, 100%), 946.4 ([2M + NH_4_]^+^, 49%), HRMS (ES^+^) calc. for C_26_H_29_N_2_O_6_ [M + H]^+^ *m/z* 465.2020, found 465.2007.

### 4.4. Preparation of 3,5′-Dibenzyl–uridine (**11**)

Compound **10** (1.1 g, 2.58 mmol) was added in a 1/1 solution of 90% aqueous trifluoroacetic acid/DCM (25 mL) and the mixture was stirred for 4 h at RT. The reaction was monitored by TLC (hexane/acetone: 2/1) and was stopped when it was judged complete. The mixture was concentrated under vacuum and coevaporated with toluene (3 × 50 mL). The residue was purified by flash column chromatography (hexane/acetone, stepwise gradient) to give **11** as a white foam (983 mg, 90% yield). ^1^H NMR (CD_3_OD, 400 MHz) *δ* (ppm) 7.98 (d, *J* = 8.1 Hz, 1H), 7.38–7.36 (m, 4H), 7.34–7.21 (m, 6H), 5.91 (d, *J* = 4.0 Hz, 1H), 5.41 (d, *J* = 8.1 Hz, 1H), 5.04 (s, 2H), 4.61–4.53 (m, 2H), 4.18 (apt, *J* = 5.1 Hz, 1H), 4.14–4.08 (m, 2H), 3.87 (dd, *J* = 10.9, 2.4 Hz, 1H), 3.73 (dd, *J* = 10.9, 2.4 Hz, 1H). MS (ES^+^) *m/z* 425.2 ([M + H]^+^, 100%), 442.2 ([M + NH_4_]^+^, 53%), 849.3 ([2M + H]^+^, 20%), 866.4 ([2M + NH_4_]^+^, 5%), HRMS (ES^+^) calc. for C_23_H_25_N_2_O_6_ [M + H]^+^ *m/z* 425.1707, found 425.1704.

### 4.5. General Procedure for the Fluorination Reactions

In a dry, clean Teflon flask, the nucleoside (50–100 mg, 1 eq.) was dissolved in dry solvent (4–10 mL, toluene). Under cooling at −78 °C, stirring and argon atmosphere, Deoxo-Fluor (50% solution in THF) (10 eq.) was slowly added with a plastic syringe. The reaction was then left to stir at RT for an appropriate time, monitored by TLC. When complete, 3 mL of ethyl acetate were added. Then, 5 mL of calcium carbonate or sodium bicarbonate saturated solution were added dropwise. The reaction mixture was stirred at RT for 30 min. Then, the mixture was transferred into a separating funnel and the organic layer was separated. The aqueous layer was washed with ethyl acetate (5 mL). The combined organic layers were dried over MgSO_4_, evaporated under vacuum and purified by flash column chromatography (hexane/ethyl acetate: stepwise gradient).

#### 4.5.1. 1-(5-O-Benzyl-3-O-2′-deoxy-2′-fluoro–β-d-arabinofuranosyl)-N^3^-benzyluracil (**12**)

Compound **12** was prepared (54 mg, 53% yield) by following the general procedure described in Section 4.5 using compound **11** (100 mg, 0.23 mmol) and Deoxo-Fluor (1 mL, 2.36 mmol) in toluene (4 mL). mp: 131–133 °C. ^1^H NMR (CDCl_3_, 500 MHz) *δ* (ppm) 7.63 (d, *J* = 8.2 Hz, 1H), 7.48–7.44 (m, 2H), 7.40–7.27 (m, 8H), 5.92 (d, *J* = 20.9 Hz, 1H), 5.63 (d, *J* = 8.1 Hz, 1H), 5.17–5.01 (m, 2 H), 4.98 (d, *J* = 48.8 Hz, 1H), 4.61 (aq, *J* = 11.5 Hz, 2H), 4.43 (dd, *J* = 2.8, 9.9 Hz, 1H), 4.31 (td, *J* = 2.8 Hz, 1H), 4.11 (dd, *J* = 11.1, 3.5 Hz, 1H), 4.11 (dd, *J* = 11.1, 3.5 Hz, 1H). ^19^F{H}NMR (CDCl_3_, 282 MHz) *δ* (ppm) −185 (s, 1F). ^19^F NMR (CDCl_3_, 282 MHz) *δ* (ppm) −185 (ddd, *J* = 48.8, 20.9, 9.9 Hz). ^13^C NMR (CDCl_3_, 126 MHz) *δ* (ppm) 162.6, 151.1, 138.8, 136.6 (2), 129.3, 128.9, 128.7, 128.6, 128.1, 127.9, 101.9, 98.7 (d, *J* = 185.5 Hz), 91.0 (d, *J* = 38.8 Hz), 80.8, 74.8 (d, *J* = 27.4 Hz), 74.5, 68.5, 44.3. MS (ES^+^) *m/z* 427.2 ([M + H]^+^, 100%), 449.1 ([M + Na]^+^, 26%), 875.3 ([2M + Na]^+^, 15%), HRMS (ES^+^) calc. for C_23_H_24_F_1_N_2_O_5_ [M + H]^+^ *m/z* 427.1664, found 427.1662.

#### 4.5.2. 1-(5-O-Benzyl-3-O-2′,3′-dideoxy-2′,3′-difluoro-β-D-xylofuranosyl)-N^3^-benzyluracil (**13**)

Compound **13** was prepared (11.5 mg, 11% yield) by following the general procedure in Section 4.5 using compound **11** (100 mg, 0.23 mmol) and Deoxo-Fluor (1 mL, 2.36 mmol) in toluene (4 mL). ^1^H NMR (CDCl_3_, 500 MHz) *δ* (ppm) 7.51–7.45 (m, 2H), 7.41–7.27 (m, 9H), 6.13 (d, *J* = 19.5 Hz, 1H), 5.73 (d, *J* = 8.2 Hz, 1H), 5.22 (ddd, *J* = 48.7, 7.5, 2.5 Hz, 1H), 5.13 (dd, *J* = 47.8, 10.4 Hz, 1H), 5.12 (m, 2 H), 4.7 (m, 2H), 4.66–4.57 (m, 2H), 3.89 (dd, *J* = 10.2, 5.8 Hz, 1H), 3.84 (ddd, *J* = 10.2, 5.9, 1.6 Hz, 1H). ^19^F{H}NMR (CDCl_3_, 282 MHz) *δ* (ppm) −192 (d, *J* = 14.3 Hz, 1F), −208 (d, *J* = 14.3 Hz, 1F). ^13^C NMR (CDCl_3_, 126 MHz) *δ* 162.5, 151.0, 137.3, 137.0, 136.6, 129.3, 128.8, 128.6, 128.3, 120.0, 127.9, 102.5, 96.0 (dd, *J* = 187.0, 31.4 Hz), 91.9 (dd, *J* = 184.6, 30.0 Hz), 89.4 (d, *J* = 37.4 Hz), 81.1 (d, *J* = 19.2 Hz), 73.9, 65.8 (d, *J* = 8.5 Hz), 44.4. MS (ES^+^) *m/z* 429.2 ([M + H]^+^, 100%), 446.2 ([M + NH_4_]^+^, 89%), 451.1 ([M + Na]^+^, 51%) 467.1 ([M + K]^+^, 19%), 879.9 ([2M + Na]^+^, 19%), HRMS (ES^+^) calc. for C_23_H_23_F_2_N_2_O_4_ [M + H]^+^ *m/z* 429.1620, found 429.1618.

### 4.6. Modelling of the Conformers of Compound **12**

Conformational flexibility of **12** was initially assessed by Monte Carlo MMFF conformational search. For each of the 4 stereoisomers, the 2 conformers (north and south) with the lowest relative energy were subjected to DFT geometry optimization in vacuum at the B3LYP/6-31G* level of theory. All calculations were performed using Spartan’18 (Version 1.1.4, Wavefunction, CA, USA) running on a Windows 10 computer equipped with a IntelCore i7 processor at 4 GHz and 16 GB of memory.

### 4.7. Heteronuclear NMR Spectroscopy

All ^1^H,^19^F correlations were acquired using a Bruker AVIII HD 500 NMR instrument equipped with a room-temperature 5 mm BBFO+ probe (Billerica, MA, USA). The HMBC experiment was recorded using the standard Bruker pulse sequence *hmqcgpqf* that was altered by removing decoupling during acquisition and by adding ^1^H/^19^F switches. HOESY experiments were obtained by following a protocol described elsewhere [48].

### 4.8. X-Ray Crystallography

Crystal Data for C_23_H_23_N_2_O_5_F (M = 426.44 g/mol): monoclinic, space group *P*2_1_ (no. 4), *a* = 10.1992(16) Å, *b* = 9.3210(18) Å, *c* = 10.7915(16) Å, β = 92.241(7)°, V = 1025.1(3) Å^3^, Z = 2, T = 125(2) K, μ(CuKα) = 0.869 mm^−1^, Dcalc = 1.381 g/cm^3^, 11,755 reflections measured, 3077 unique (Rint = 0.0404). The final R1 was 0.0314 (I > 2σ(I)) and wR2 was 0.0816 (all data). Flack −0.13(12). CCDC 2,027,460 contains the supplementary crystallographic data for this paper. These data can be obtained free of charge via http://www.ccdc.cam.ac.uk/conts/retrieving.html (or from the CCDC, 12 Union Road, Cambridge CB2 1EZ, UK; Fax: +44 1223 336033; E-mail: deposit@ccdc.cam.ac.uk).

## Figures and Tables

**Table 1:** Spin-spin coupling constants (calculated and experimental values) for conformers A1–D2 of compound **12**. Coupling constants are shown in Hz. Experimental values were recorded in CDCl_3._

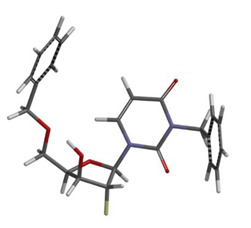							
C1 (*RS*-north)							
	Relative E (kJ/mol)	*J*_F-H1′_ (Hz)	*J*_F-H3′_ (Hz)	*J*_H1′-H2′_ (Hz)	*J*_H2′-H3′_ (Hz)	*J*_H3′-H4′_ (Hz)	Avg. Deviation
A1 (*SR*-north)	0	3.7	18.0	7.2	6.3	8.6	4.48
A2 (*SR*-south)	2.58	32.9	18.7	1.9	0.9	1.9	3.00
B1 (*RR*-north)	0	20.0	39.9	0.8	3.4	8.6	6.21
B2 (*RR*-south)	9.47	16.9	3.2	6.1	5.8	1.0	2.31
C1 (*RS*-north)	0	22.8	17.7	0.8	1.2	3.2	1.68
C2 (*RS*-south)	31.44	20.0	24.5	4.2	6.2	7.3	3.46
D1 (*SS*-north)	9.83	6.2	3.5	7.2	6.4	2.7	3.77
D2 (*SS*-south)	0	24.1	39.2	3.6	3.8	7.5	6.11
Experimental		21.2	9.6	0	0	2.8	

**Table 2:** Calculated and experimental distance values for conformers A1–D2 of compound **12**. Distances are shown in Å. Experimental values were recorded in CDCl_3._

	d(F-H2′) (Å)	d(F-H4′) (Å)	d(F-H1′) (Å)	d(F-H3′) (Å)	Avg. Deviation
A1 (*SR*-north)	2.017	4.150	3.025	2.511	0.541
A2 (*SR*-south)	2.038	4.006	3.300	2.496	0.534
B1 (*RR*-north)	2.028	2.757	2.452	3.292	0.289
B2 (*RR*-south)	2.025	4.292	2.497	2.917	0.554
C1 (*RS*-north)	2.036	2.630	2.419	2.511	0.048
C2 (*RS*-south)	2.025	4.313	2.462	2.417	0.536
D1 (*SS*-north)	2.014	4.076	3.108	2.951	0.552
D2 (*SS*-south)	2.017	4.046	3.285	3.288	0.618
NOE	1.000	0.174	0.437	0.330	
Distance	2.027	2.713	2.327	2.438	

## Supplementary Materials

The following are available online. Scheme S1: The four different stereochemistries found in the sugar moiety of the nucleosides. Figure S1: Partial ^19^F{H}NMR (top) and ^19^F NMR (bottom) of compound **12** (282 MHz, CDCl_3_). Figure S2: ^1^H{F}NMR (top) and ^1^H NMR (bottom) of compound **12** (500 MHz, CDCl_3_). Figure S3: Partial ^19^F{H}NMR of compound **13** (282 MHz, CDCl_3_). Figure S4: ^1^H NMR (top) and ^1^H{F}NMR (bottom) of compound **13** (500 MHz, CDCl_3_). Figure S5: ^1^H,^19^F HMBC of compound **13** (CDCl_3_). Figure S6: Modelling of the conformers of compound **12** (B3LYP/6-31G* level of theory). Table S1: Experimental spin-spin coupling constant values for compounds S1 and S2. Figure S7: ^1^H NMR (CD3OD, 500 MHz) of 2′,3′-*O*-isopropylidene uridine (**9**). Figure S8: ^13^C NMR DEPTQ (CD3OD, 126 MHz) spectrum of 2′,3′-*O*-isopropylidene uridine (**9**). Figure S9: HRMS of 2′,3′-*O*-isopropylidene uridine (**9**). Figure S10: ^1^H NMR (CDCl_3_, 500 MHz) of 3,5′-dibenzyl-2′,3′-*O*-isopropylidene uridine (**10**). Figure S11: DEPT-Q NMR of 3,5′-dibenzyl-2′,3′-*O*-isopropylidene uridine (**10**). Figure S12: HRMS of 3,5′-dibenzyl-2′,3′-*O*-isopropylidene uridine (**10**). Figure S13: ^1^H-NMR (CD3OD, 400 MHz) of 3,5′-dibenzyl–uridine (**11**). Figure S14: HRMS of 3,5′-dibenzyl-uridine (**11**). Figure S15: ^13^C NMR (CDCl_3_, 126 MHz) of 1-(5-*O*-benzyl-3-*O*-2′-deoxy-2′-fluoro–β-d-arabinofuranosyl)-*N*^3^-benzyluracil (**12**). Figure S16: HRMS of 1-(5-*O*-benzyl-3-*O*-2′-deoxy-2′-fluoro–β-d-arabinofuranosyl)-*N*^3^-benzyluracil (**12**). Figure S17: ^13^C NMR (CDCl_3_, 126 MHz) of 1-(5-*O*-benzyl-3-*O*-2′,3′-dideoxy-2′,3′-difluoro-β-d-*xylo*furanosyl)-*N*^3^-benzyluracil (**13**). Figure S18: HRMS of 1-(5-*O*-benzyl-3-*O*-2′,3′-dideoxy-2′,3′-difluoro-β-d-*xylo*furanosyl)-*N*^3^-benzyluracil (**13**).
