# Supplementary material for: Synthesis and Conformational Analysis of Fluorinated Uridine Analogues Provide Insight into a Neighbouring-Group Participation Mechanism
Source: Molecules. 2020 Nov 25;25(23):5513. doi: 10.3390/molecules25235513 (PMC7728060; doi:10.3390/molecules25235513)
Supplement: Supplementary file 1 [file molecules-25-05513-s001.pdf]

Supporting Information

Synthesis and conformational analysis of fluorinated uridine analogues provide insight into a neighbouring group participation mechanism

Freideriki Michailidou[1,2], Tomas Lebl[1], Alexandra M. Z. Slawin[1], Sunil Vishnuprasadji Sharma,[1] Murray J. B. Brown[2], and Rebecca Jane Miriam Goss[1*]

[1] School of Chemistry, University of St Andrews, North Haugh, St Andrews, Fife, KY16 9ST, UK; rjmg@st-andrews.ac.uk

[2] GSK, Stevenage, SG1 2NY, UK

* Correspondence: rjmg@st-andrews.ac.uk; Tel.: (optional; include country code; if there are multiple corresponding authors, add author initials) +xx-xxxx-xxx-xxxx (F.L.)

Received: date; Accepted: date; Published: date

Table of contents

Scheme S1 3

Analysis of ^{19}F and ^{1}H NMR spectra of compounds 12 and 13 3

Figure S1 3

Figure S2. 4

Figure S3. 5

Figure S4 5

Figure S5 6

Conformational analysis of compounds 12-13 and related literature data 7

Figure S6 7

Table S1. 8

Figure S7 9

Figure S8..9
Figure S9..10
Figure S10..10
Figure S12..11
Figure S13..12
Figure S14..12
Figure S15..13
Figure S16..13
Figure S17..14
Figure S18..14

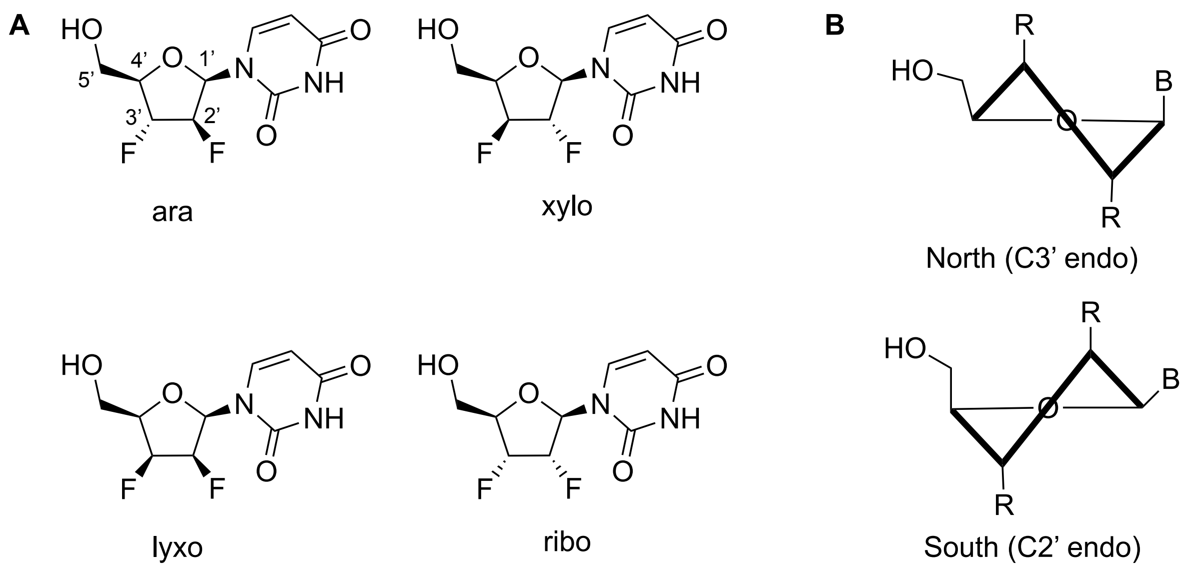

Scheme S1. A The four different stereochemistries found in the sugar moiety of the nucleosides: 'ribo', 'ara', 'xylo' and 'lyxo'. The sugar moiety is numbered to indicate the relevant positions. **B** The 'North' and 'South' conformation of nucleosides.

Analysis of ^{19}F and ^{1}H NMR spectra of compounds 12 and 13

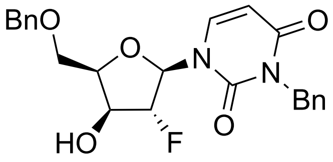

12

The desired monofluorinated product **12**, obtained in a 53% yield after flash column chromatography, was analysed by NMR. The ^{19}F{H}NMR revealed a singlet at –185 ppm. The proton coupled spectrum revealed a ddd, with the expected coupling constants: $^{2}J_{F\text{-}H2'}$ 48.8 Hz, $^{3}J_{F\text{-}H1'}$ 20.9 Hz, $^{3}J_{F\text{-}H3'}$ 9.9 Hz (**Figure S**1).

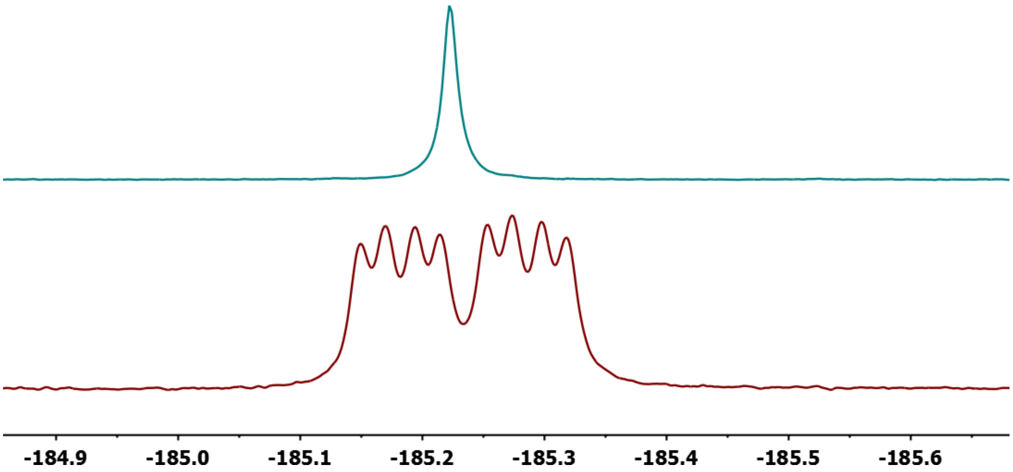

Figure S1. Partial ^{19}F{H}NMR (top) and ^{19}F NMR (bottom) of compound **12** (282 MHz, $CDCl_3$).

The position of the fluorine at the C2′ was verified by ^{1}H NMR and $^{1}H\{F\}$NMR. The apparent singlet at 5.92 ppm in the fluorine decoupled ^{1}H NMR corresponds to the H1′, and splits into a doublet with a coupling constant of 20.9 Hz in the fluorine coupled spectrum. This coupling constant is representative of ^{3}J proton-fluorine interaction (**Figure S2**).

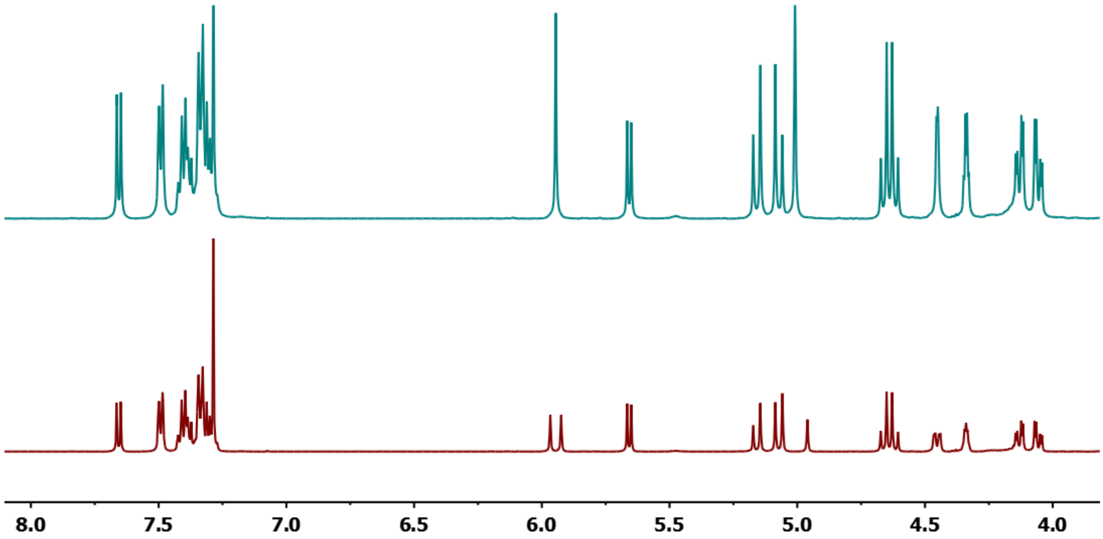

Figure S2. $^{1}H\{F\}$NMR (top) and ^{1}H NMR (bottom) of compound **12** (500 MHz, $CDCl_3$).

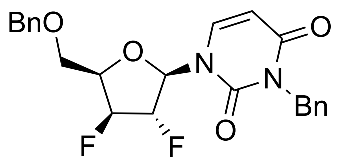

The $^{19}F\{H\}$NMR (**Figure S3**) of the compound assigned as the difluorinated molecule **13**, revealed the expected doublets at –192 and –208 ppm (J = 14.3 Hz).

13

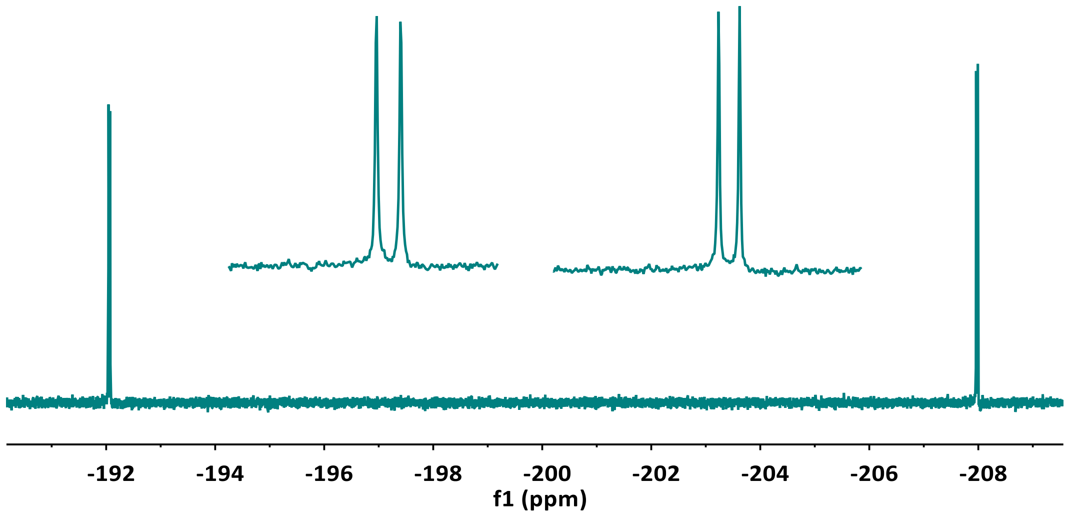

Figure S3. Partial $^{19}F\{H\}$NMR of compound **13** (282 MHz, $CDCl_3$).

The ^{1}H NMR and $^{1}H\{F\}$NMR of compound **13** were recorded. The H4′ appears as a td of 1 proton at 4.50 ppm in the $^{1}H\{F\}$NMR, but splits into two multiplets of 0.5 protons each in the ^{1}H NMR (**Figure S4**).

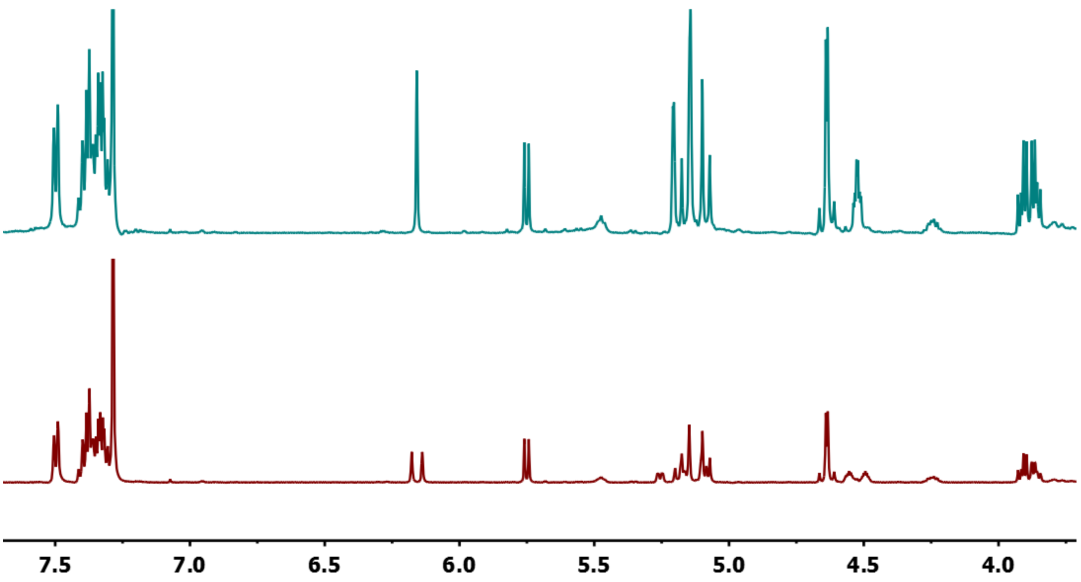

Figure S4. ^{1}H NMR (top) and $^{1}H\{F\}$NMR (bottom) of compound **13** (500 MHz, $CDCl_3$).

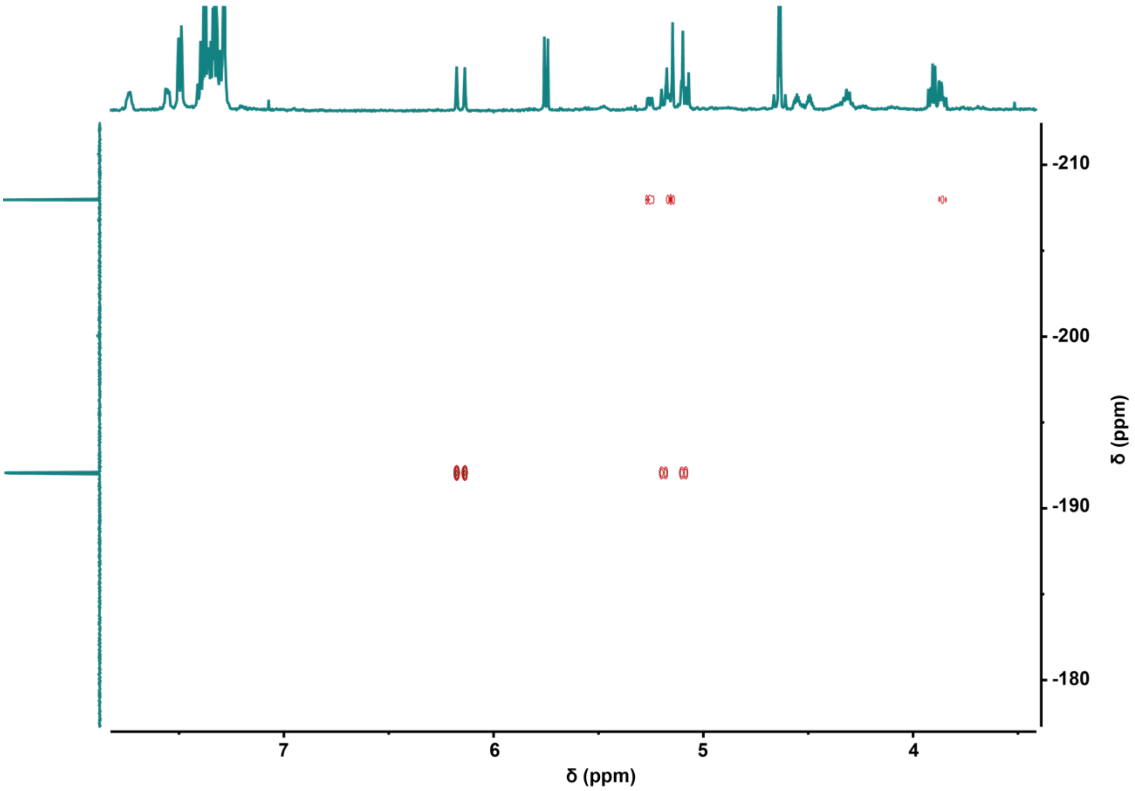

Figure S5. C ^{1}H,^{19}F HMBC of compound **13** ($CDCl_3$).

Conformational analysis of compounds 12-13 and related literature data

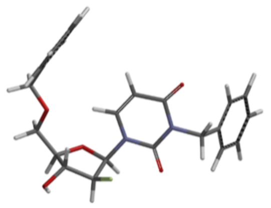

A1 (SR-north)

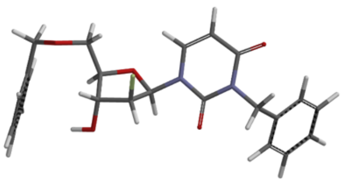

A2 (SR-south)

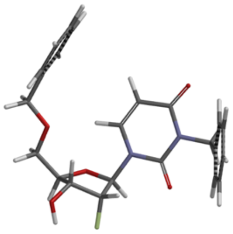

B1 (RR-north)

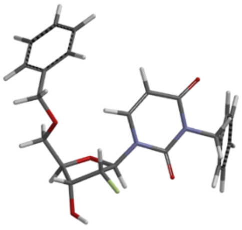

B2 (RR-south)

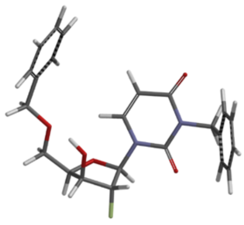

C1 (RS-north)

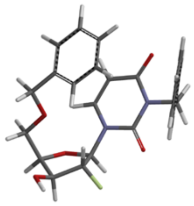

C2 (RS-south)

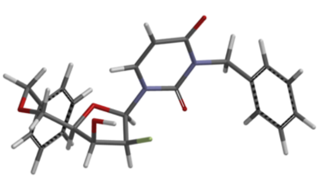

D1 (SS-north)

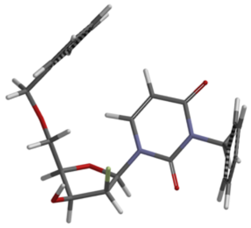

D2 (SS-south)

Figure S6. Modelling of the conformers of compound **12** (B3LYP/6-31G* level of theory).

Barchi *et al.*[23] reported the synthesis and conformational analysis of the 2′,3′-difluoro-dideoxy uridine analogues with the ara- and xylo- stereochemistry (Table S1). The coupling constants values are in agreement with McAtee *et al.*[22]

Table S1. Experimental spin-spin coupling constant values for compounds S1 and S2, adapted from Barchi *et al.*[23] Coupling constants are shown in Hz.

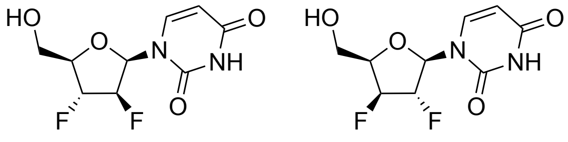

S1 **S2**

Compound	T(K)	$J_{H1'-H2'}$	$J_{H2'-H3'}$	$J_{H3'-H4'}$
**S1**	283	3.57	1.63	3.33
	343	3.76	1.72	3.48
**S2**	283	1.07	1.05	2.58
	343	1.65	1.18	2.69

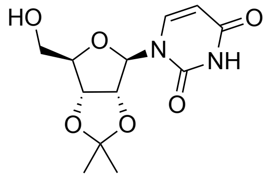

9

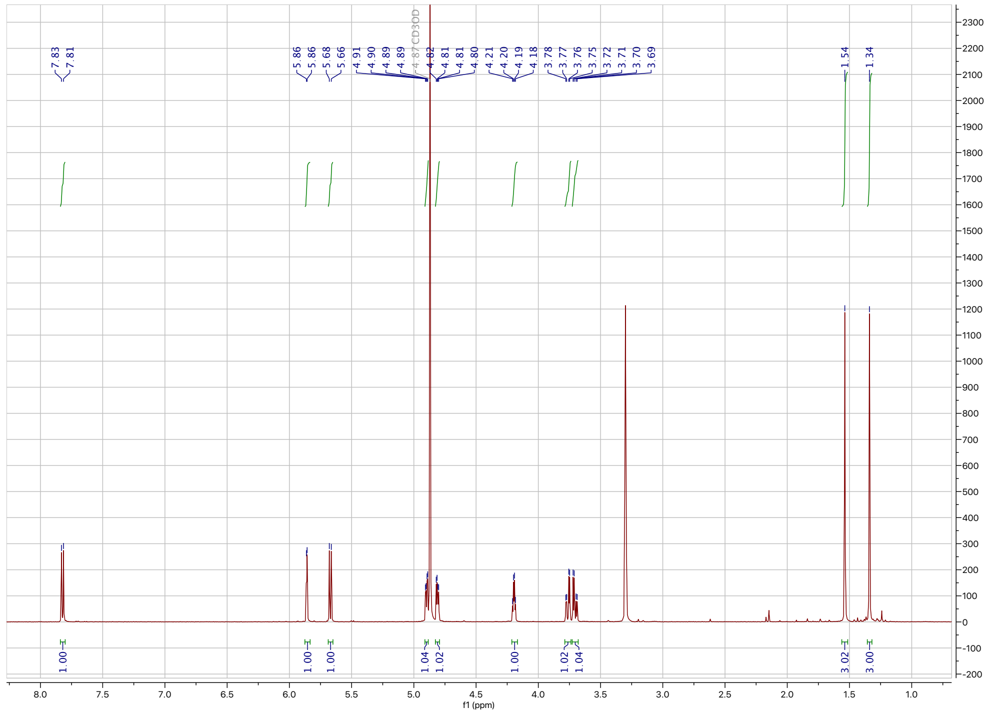

Figure S7. ^{1}H NMR (CD_3OD, 500 MHz) of 2′,3′-*O*-isopropylidene uridine (9).

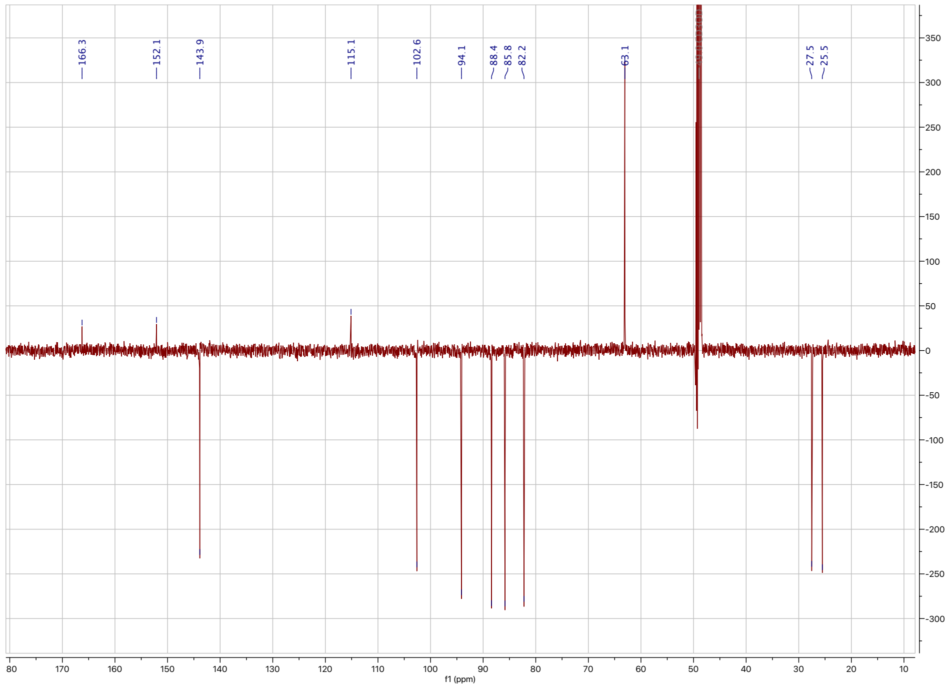

Figure S8. ^{13}C NMR DEPTQ (CD_3OD, 126 MHz) spectrum of 2′,3′-*O*-isopropylidene uridine (9).

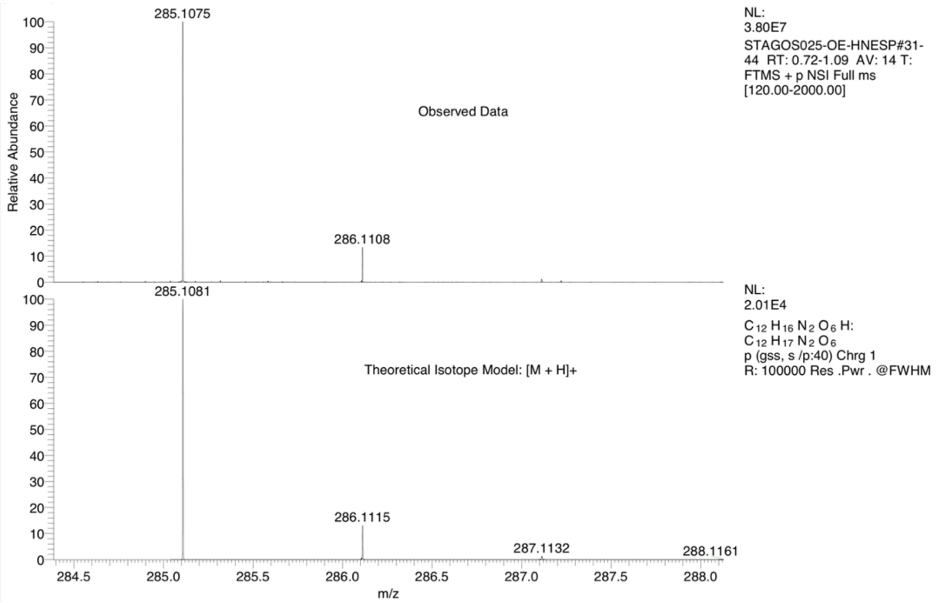

Figure S9. HRMS of 2′,3′-*O*-isopropylidene uridine (9). HRMS (ES+) *m*/*z* calc. for $C_{12}H_{17}N_2O_6$ $[M + H]^+$ 285.1081, found 285.1075.

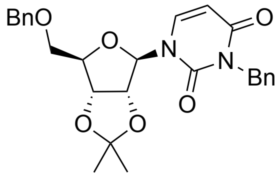

10

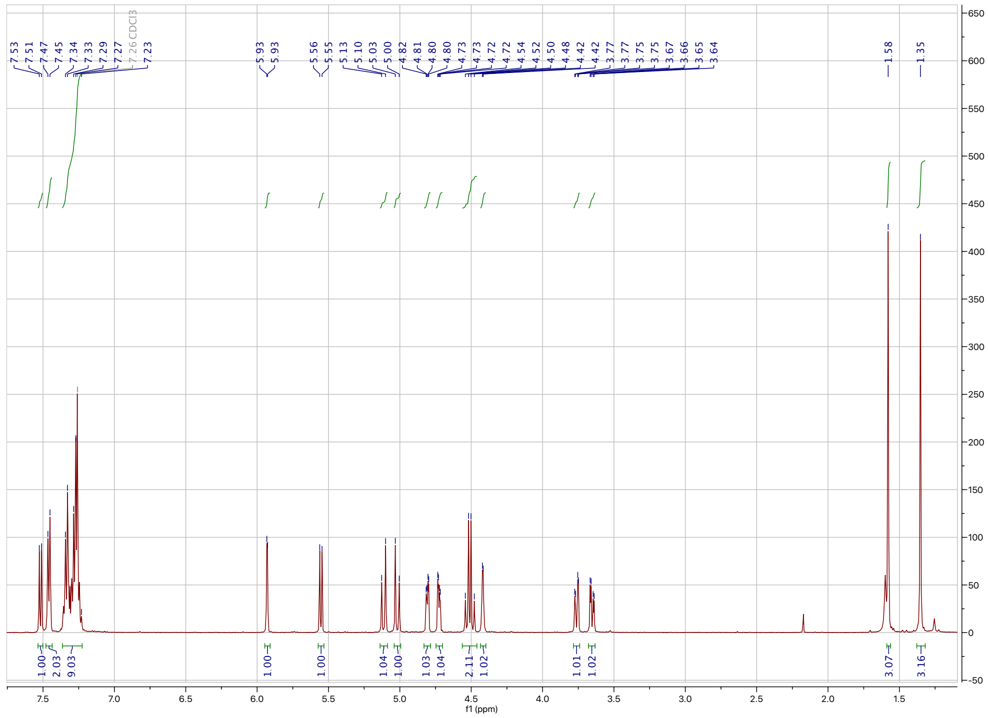

Figure S10. ^1H NMR ($CDCl_3$, 500 MHz) of 3,5′-dibenzyl-2′,3′-*O*–isopropylidene uridine (10).

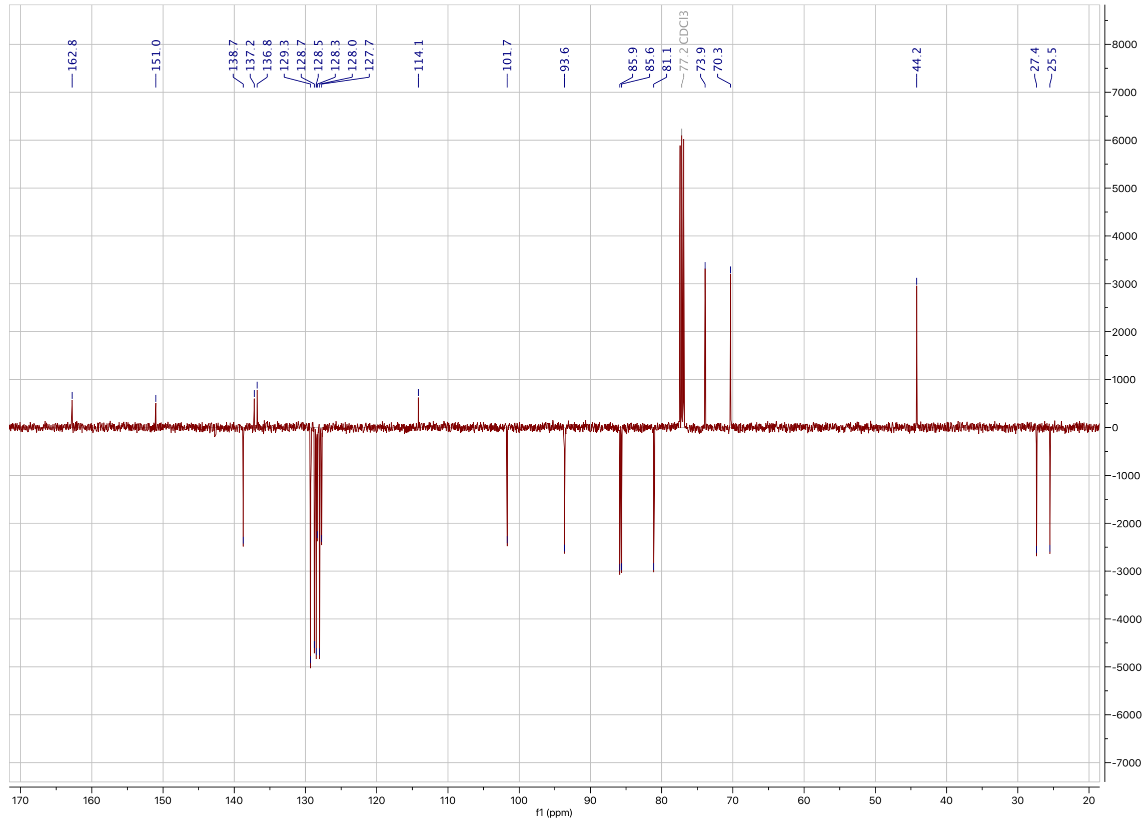

Figure S11. ^{13}C NMR DEPTQ NMR of 3,5′-dibenzyl-2′,3′-*O*–isopropylidene uridine (10).

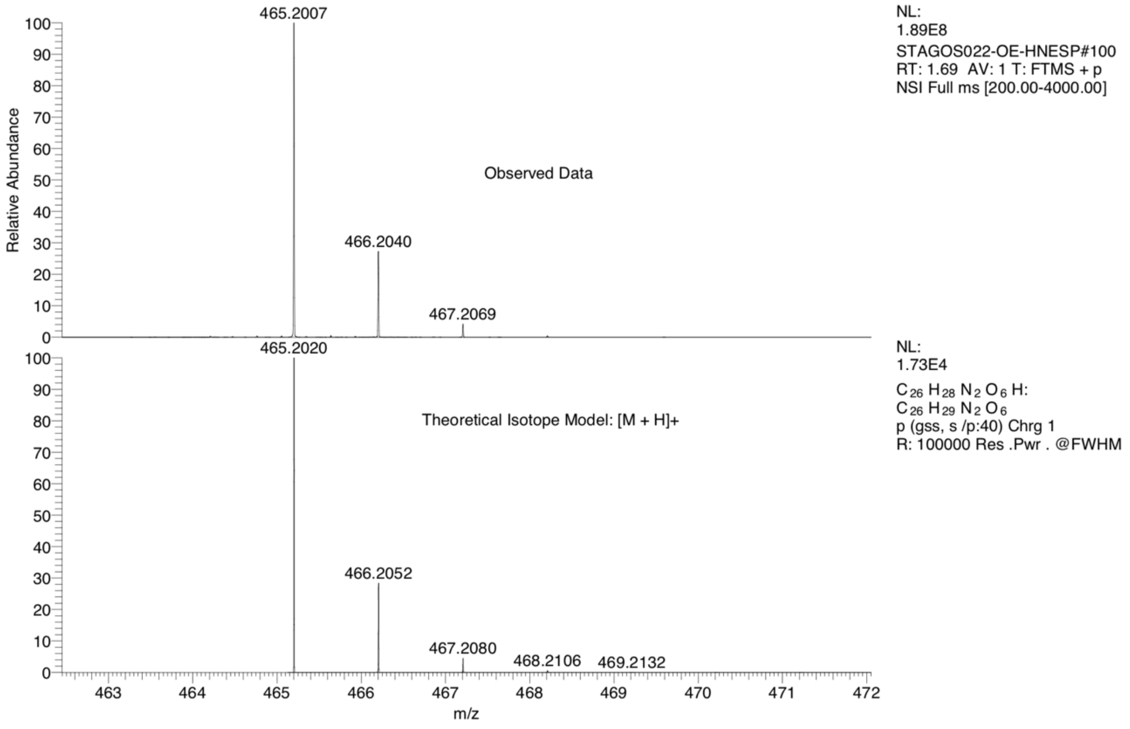

Figure S12. HRMS of 3,5′-dibenzyl-2′,3′-*O*–isopropylidene uridine (10).

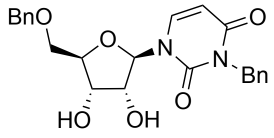

11

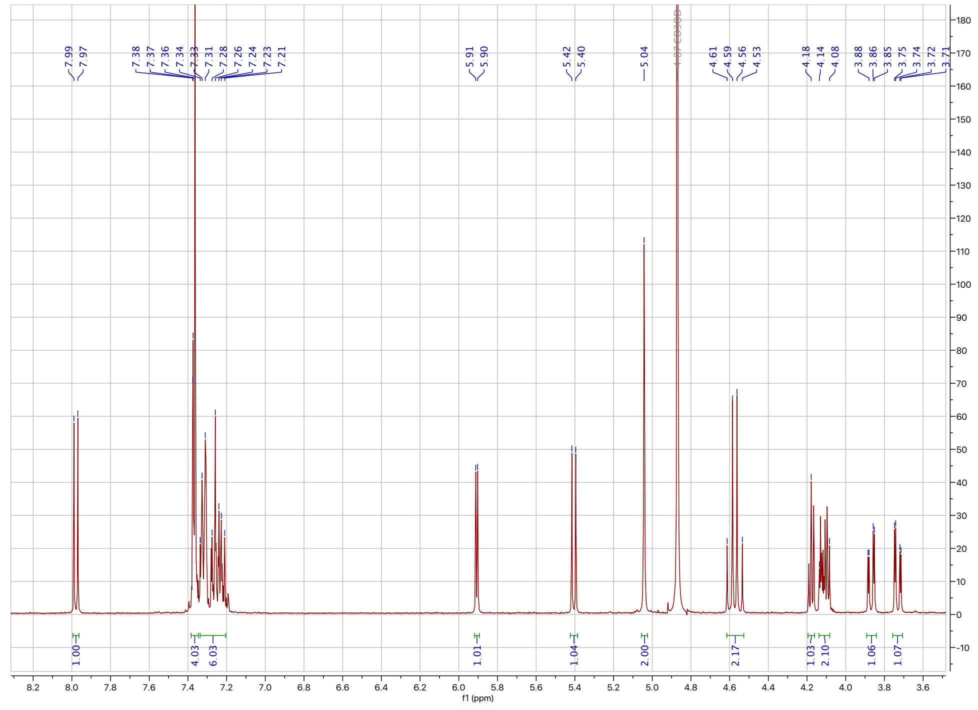

Figure S13. ^{1}H-NMR (CD_3OD, 400 MHz) of 3,5′-dibenzyl–uridine (11).

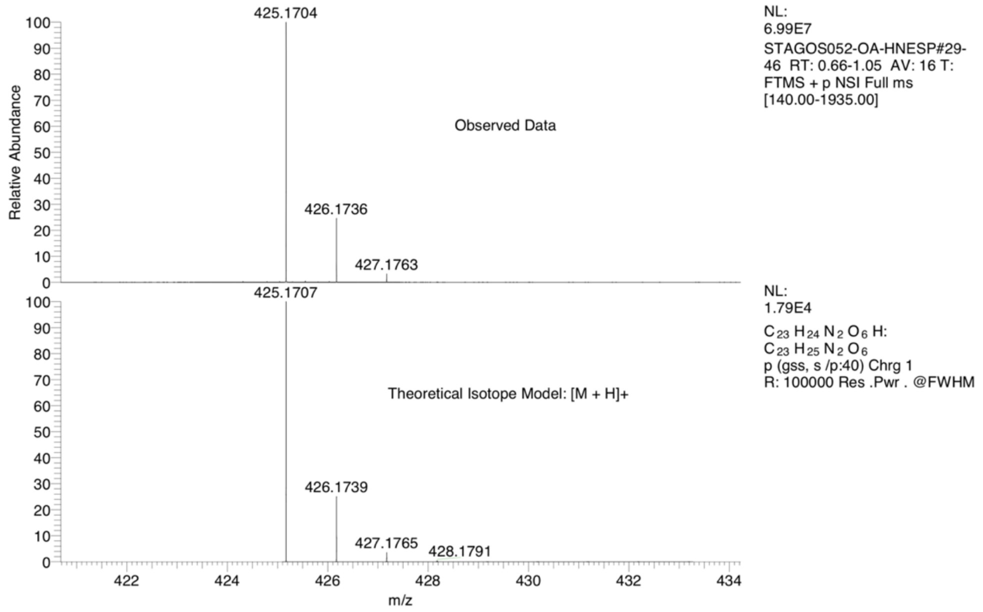

Figure S14. HRMS of 3,5′-dibenzyl–uridine (11). HRMS (ES^{+}) calc. for $C_{23}H_{25}N_2O_6$ [M + H]$^{+}$ *m/z* 425.1707, found 425.1704.

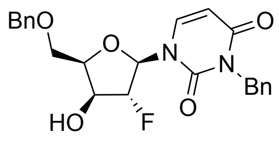

12

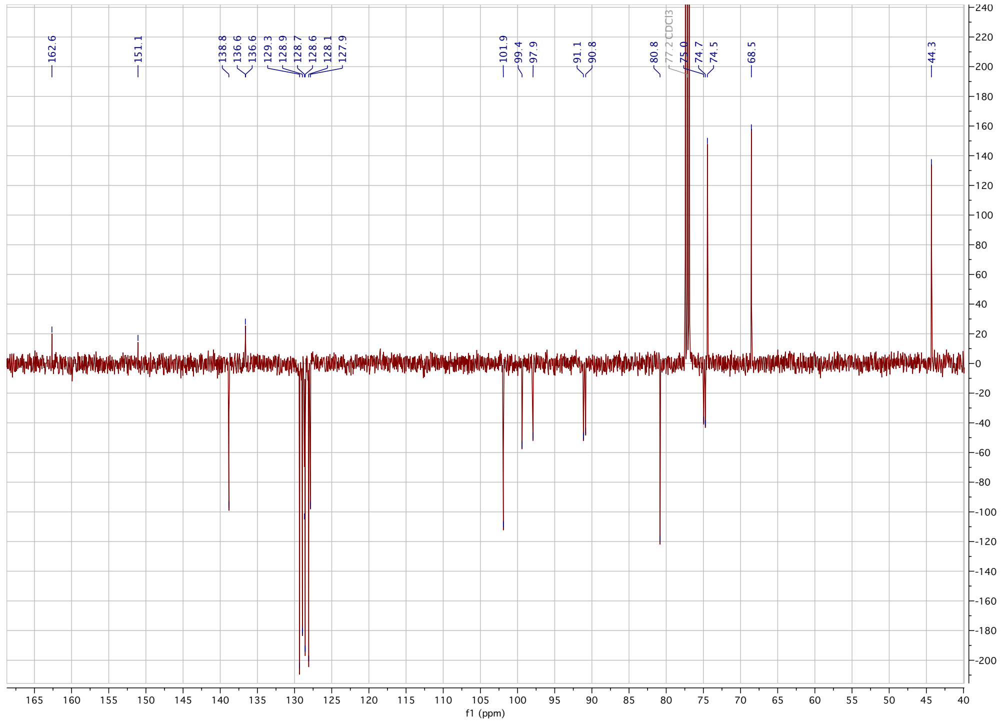

Figure S15. ^{13}C NMR ($CDCl_3$, 126 MHz) of 1-(5-*O*-benzyl-3-*O*-2′-deoxy-2′-fluoro–*β*-D-arabinofuranosyl)-*N*^{3}-benzyluracil (12).

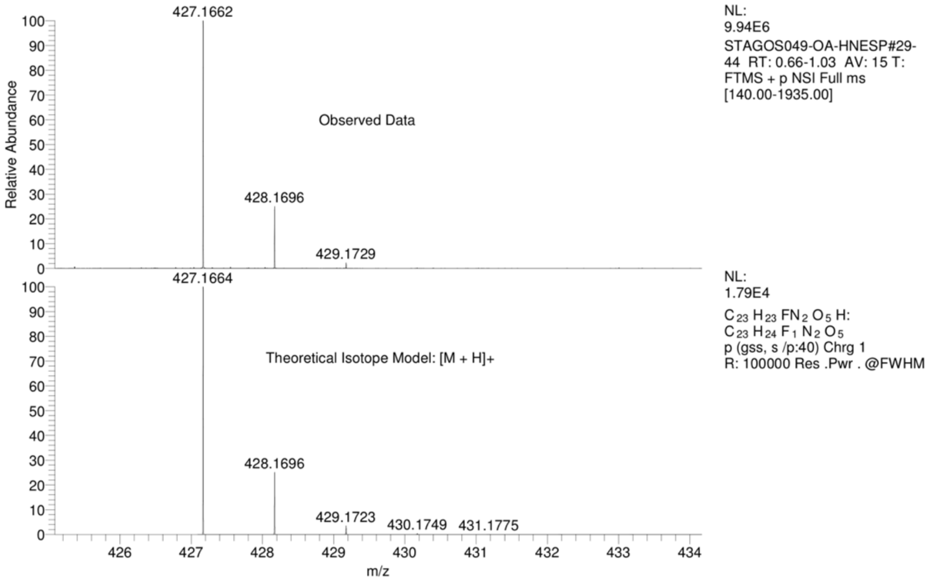

Figure S16. HRMS of 1-(5-*O*-benzyl-3-*O*-2′-deoxy-2′-fluoro–*β*-D-arabinofuranosyl)-*N*^{3}-benzyluracil (12). HRMS (ES$^+$) calc. for $C_{23}H_{24}F_1N_2O_5$ [M + H]$^+$ *m*/*z* 427.1664, found 427.1662.

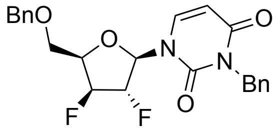

13

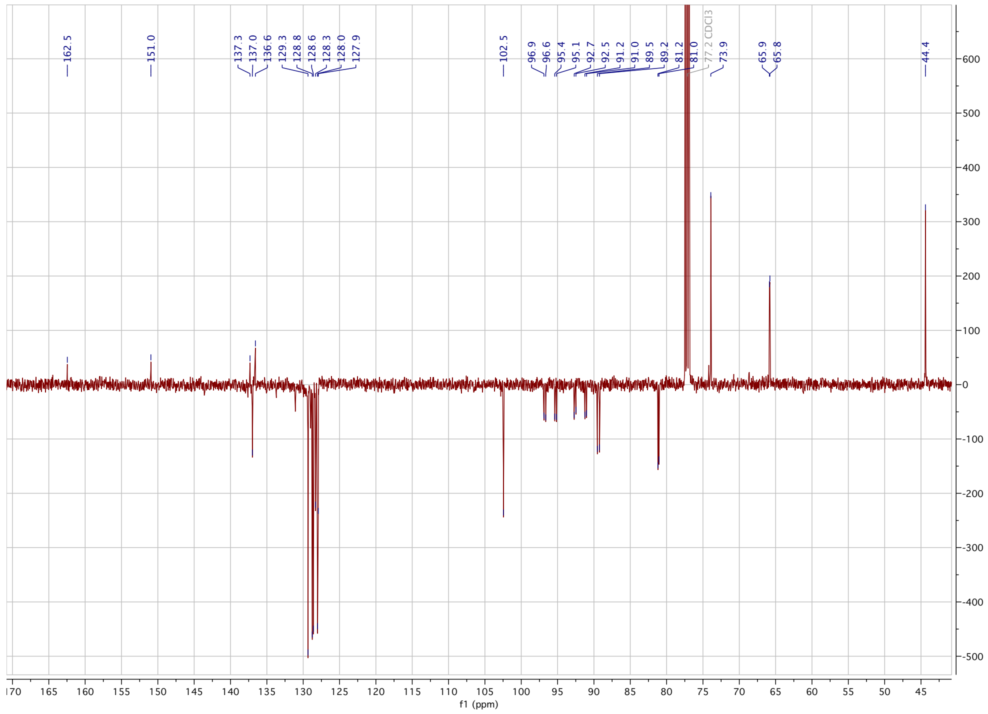

Figure S17. ^{13}C NMR ($CDCl_3$, 126 MHz) of 1- (5-*O*-benzyl-3-*O*-2′,3′-dideoxy-2′,3′-difluoro-β-D-xylofuranosyl)-N^3-benzyluracil (13).

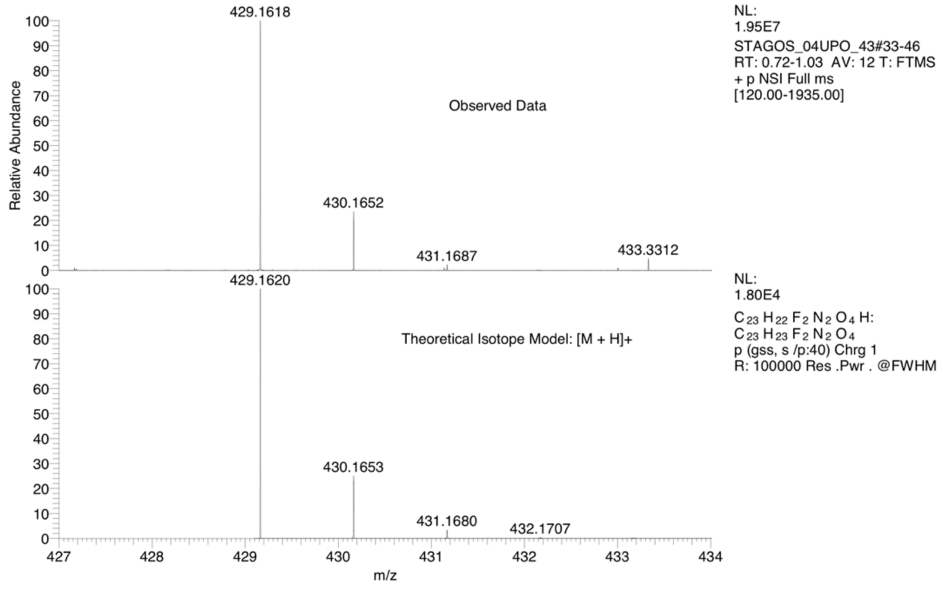

Figure S18. HRMS of 1- (5-*O*-benzyl-3-*O*-2′,3′-dideoxy-2′,3′-difluoro-β-D-xylofuranosyl)-N^3-benzyluracil (13). HRMS (ES^+) calc. for $C_{23}H_{23}F_2N_2O_4$ $[M + H]^+$ *m/z* 429.1620, found 429.1618.
